# Lung Ultrasound and Clinical Progression of Acute Bronchiolitis: A Prospective Observational Single-Center Study

**DOI:** 10.3390/medicina56060314

**Published:** 2020-06-26

**Authors:** Antonio Di Mauro, Anna Rita Cappiello, Angela Ammirabile, Nicla Abbondanza, Francesco Paolo Bianchi, Silvio Tafuri, Mariano M. Manzionna

**Affiliations:** 1 Department of Biomedical Science and Human Oncology, “Aldo Moro” University of Bari, 70100 Bari, Italy; dimauroantonio@msn.com (A.D.M.); a.ammirabile4@studenti.uniba.it (A.A.); frapabi@gmail.com (F.P.B.); silvio.tafuri@uniba.it (S.T.); 2 U.O.C. Pediatric and Neonatology, San Paolo Hospital, ASL BARI, 70100 Bari, Italy; annaritacappiello@yahoo.it (A.R.C.); niclabbondanza@gmail.com (N.A.)

**Keywords:** lung ultrasound, bronchiolitis, infants

## Abstract

*Background and Objectives:* Recent literature suggests that lung ultrasound might have a role in the diagnosis and management of bronchiolitis. The aim of the study is to evaluate the relationship between an ultrasound score and the clinical progression of bronchiolitis: need for supplemental oxygen, duration of oxygen therapy and hospital stay. *Materials and Methods:* This was a prospective observational single-center study, conducted in a pediatric unit during the 2017–2018 epidemic periods. All consecutive patients admitted with clinical signs of acute bronchiolitis, but without the need for supplemental oxygen, underwent a lung ultrasound in the first 24 h of hospital care. The lung involvement was graded based on the ultrasound score. During clinical progression, need for supplemental oxygen, duration of oxygen therapy and duration of hospital stay were recorded. *Results:* The final analysis included 83 patients, with a mean age of 4.5 ± 4.1 months. The lung ultrasound score in patients that required supplemental oxygen during hospitalization was 4.5 ± 1.7 (range: 2.0–8.0), different from the one of the not supplemented infants (2.5 ± 1.8; range: 0.0–6.0; *p* < 0.001). Ultrasound score was associated with the need for supplemental oxygen (OR = 2.2; 95% CI = 1.5–3.3; *p* < 0.0001). Duration of oxygen therapy was not associated with LUS score (*p* > 0.05). Length of hospital stay (coef. = 0.5; 95% CI = 0.2–0.7; *p* < 0.0001) correlates with LUS score. *Conclusion:* Lung ultrasound score correlates with the need of supplemental oxygen and length of hospital stay in infants with acute bronchiolitis.

## 1. Introduction

Acute bronchiolitis is a viral infection of the lower respiratory tract. Respiratory syncytial virus is the most frequent cause of bronchiolitis and epidemiological data showed that it infects 90% of infants and young children during the first two years of life [1]. Bronchiolitis represents the first cause of hospitalization in the first 12 months of life in the developed countries, responsible for approximately 100,000 admissions every year. For this reason, we need new strategies to safely reduce bronchiolitis hospitalizations [2].

According to the recent NICE and American Academy of Pediatrics clinical guidelines, the diagnostic algorithm of bronchiolitis includes just clinical history and physical examination. Radiographic or laboratory studies should not be obtained routinely and restricted only to complicated cases [3,4].

Despite evidence-based guidelines, chest X-rays (CXR) represents the most commonly performed test in clinical practice. There is an emerging need to reduce its use to prevent exposure to radiation and useless prescription of antibiotics [5].

In the last decade, several studies focused on the definition of criteria for hospital admission, to classify affected children into a high-risk group in need for oxygen supplementation and a low-risk one that can be safely managed in an outpatient setting [6]. In this way, either hospitalization or bronchiolitis costs could be reduced.

Furthermore, some authors speculate that point-of-care lung ultrasound (LUS) could have a critical role in young children with respiratory tract infections and wheeze [7].

In 2015, Basile et al. studied the association between lung ultrasound findings and subsequent need of hospitalization and oxygen supplementation. They built a LUS score to identify infants in need for oxygen supplementation with high specificity [8].

The primary aim of the study was to evaluate the correlation between lung ultrasound score in the first 24 h of hospital stay, and the need of oxygen supplementation within the first 24 h after LUS examination. As the secondary endpoint of this study, we considered the correlation between lung ultrasound score and the length of oxygen supplementation and hospital stay; the correlation between lung ultrasound score and clinical score during the first 24 h of hospital stay and at discharge.

## 2. Methods

This was a prospective observational single-centre study, conducted in a pediatric unit during the 2017–2018 epidemic periods. We enrolled all patients aged from 0 to 24 months admitted with clinical signs of acute bronchiolitis.

On the basis of history and physical evaluation, diagnostic criteria for admission were: (1) onset of rhinorrhoea and/or upper respiratory tract infections ≤ 3 days, (2) crackles and/or wheezing, (3) use of accessory muscles or lower chest wall retractions, (4) O_2_ saturation level ≤ 96%, (4) high respiratory rate relative to age, (5) nasal flaring, (6) fever and (7) ≤75% of normal feeds. Three or more diagnostic criteria were needed to include patients.

Infants with supplemental oxygen requirement at admission were excluded from the study.

During the first 24 h of hospital stay, the severity of bronchiolitis was assessed by the attending physician according to a previous score published by Liu et al. [9], conveniently modified and already used by Basile et al. [8] (Table 1). At the same time, chest ultrasound scans were acquired by an expert paediatric sonographer (M. Manzionna, ESAOTE MyLab™Seven, Linear Probe with frequency range of 3–13 MHz) unaware of the previously determined clinical score, in order to widen the clinical evaluation. Unavailability of the pediatric sonographer after the clinical score evaluation was considered an exclusion criterium.

The lung involvement was graded on the basis of the ultrasound score, previously published and validated by Basile et al. [8] that allowed an assessment of the severity of bronchiolitis: normal LUS pattern—score 0; mild bronchiolitis—score 1–3; moderate bronchiolitis—score 4–6; and severe bronchiolitis—score 7–8 (Table 2).

The employed LUS methodology was previously described by others, placing the probe perpendicular, oblique and parallel to the ribs in the anterior (between sternum and anterior axillary line), lateral (between anterior and posterior axillary line) and posterior (between posterior axillary line and spine, lower and upper) thorax [10,11]. In this way, both longitudinal and transversal sections could be collected.

**Figure 1:**
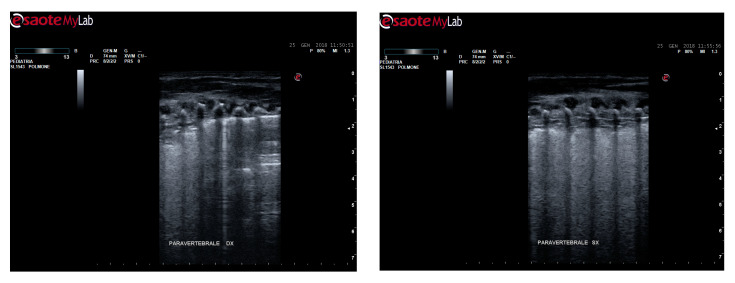
Confluent B-lines and sub-pleural consolidations in the paravertebral area scans.

The scanning of the posterior/paravertebral area was particularly important to ascertain the severity of bronchiolitis with a higher accuracy, considering the major involvement of the region for the effect of gravity on the supine position of infants (Figure 1).

According to Varshney et al., a negative LUS among children with suspected respiratory tract infections and wheeze, seems to rule in a diagnosis of severe bronchospasm instead of an episode of bronchiolitis [7]. For this reason, children with a negative LUS pattern have been diagnosed with asthma and excluded from the final analysis.

The attending physicians, unaware of the LUS scores, decided the need for supplemental oxygen. According to our hospital routine, oxygen supplementation was considered necessary to maintain saturation > 92% or capillary blood oxygen tension > 45 mmHg. High-flow nasal cannula was used as a non-invasive ventilation technique, considering its advantages in the delivery of humidified and heated air, generation of a small continuous positive airway pressure (CPAP) effect and reduction in the working of breathing [12].

We also recorded the duration of oxygen therapy and hospital stay during clinical progression.

The LUS assessment of patients was continued until all the abnormal findings disappeared and a final LUS and clinical score were recalculated at discharge.  

According to our protocol, criteria for discharge were: (1) O_2_ saturation levels > 96% at ambient air, (2) absence of dyspnea, and (3) adequate oral intake of fluids and feeds (>75%).

### Statistical Analysis

The collected data were uploaded as an Excel file and data were analysed by the Stata MP15 software.

Continuous variables were described as mean ± standard deviation and range, categorical variables as proportions.

The skewness and kurtosis test were used to evaluate the normality of the continuous variables and a normalization model was set to normalize the not normally distributed variables.

The t student test for independent data (parametric) was used to compare continuous variables between groups.

To evaluate the correlation between the US score and the clinical score (during the first 24 h from admission and at discharge), the Spearman rank’s correlation test was used: the Spearman’s rho, with a 95% confidence interval (95%CI), was estimated. The strength of the correlation is defined by the absolute value of Spearman’s rho as follows:

- 0.00–0.19 → “very weak”;
- 0.20–0.39 → “weak”;
- 0.40–0.59 → “moderate”;
- 0.60–0.79 → “strong”;
- 0.80–1.00 → “very strong”.

The association between the need for supplemental oxygen within 24 h after the ultrasound was performed (YES/NO; dependent variable) and the ultrasound score (independent variable) was assessed by the univariate logistic regression analysis. The odds ratio (OR) value, with 95%CI, the standard error, the z-test and the *p*-value were estimated.

The association between the ultrasound score in the first 24 h (dependent variable) and the duration of oxygen therapy (days; independent variable) and hospital stay (days; independent variable) was assessed by the univariate linear regression analysis. The correlation coefficient values, with 95%CI, the standard error, the *t*-test, the *p*-value and the coefficient of multiple determination (R2) were estimated.

For all the tests, a two-sided *p*-value < 0.05 was considered statistically significant.

## 3. Results

A total of 111 infants were admitted for suspected bronchiolitis. A total of 20 children were excluded shortly after their hospital admission due to supplemental oxygen requirement at admission and lack of clinical and ultrasound scores.

The previously described clinical score allowed to stratify the remaining 92 children in: healthy infant (n. 2), or mild (n. 70), moderate (n. 19) or severe (n. 1) cases of bronchiolitis.

Furthermore, the diagnosis and severity of bronchiolitis were assessed according to the US score performed by the sonographer as follows: 52 patients were diagnosed with a mild bronchiolitis, 35 patients with a moderate bronchiolitis and 2 patients with a severe bronchiolitis. A total of 8 patients with a normal LUS pattern were excluded and subsequently diagnosed with asthma (n. 6) or with an in-hospital admission referable to family concern (n. 2).

The final analysis included 84 patients diagnosed with bronchiolitis, with a mean age of 4.5 ± 4.1 months (range: 1.0–18.0; median: 3.0 month). Results are shown in Table 3.

Taking these data into consideration, it was possible to assert that the ultrasound score was weakly correlated with the clinical score at admission (rho = 0.23; 95%CI = 0.02–0.43; *p* = 0.036).

On the basis of our protocol on bronchiolitis, 27 infants (32.5%) needed oxygen supplementation during hospitalization. The mean length of oxygen supplementation was 74 h.

The LUS score was 4.5 ± 1.7 (range: 2.0–8.0) in patients that required supplemental oxygen, different from the one of the not supplemented infants (2.5 ± 1.8; range: 0.0–6.0; *p* < 0.001).

While the need for supplemental oxygen (OR = 2.2; 95% CI = 1.5–3.3; standard error = 0.45; z = 3.9; *p* < 0.0001) seemed to be associated with the ultrasound score, the duration of oxygen supplementation was not associated (coef. = 0.01; 95%CI = −0.01–0.03; standard error = 0.01; t = 0.9; *p* = 0.363; R2 = 0.03).

In our study, none of the enrolled children experienced non-invasive ventilation (NIV) failure or the need of Pediatric Intensive Care Unit (PICU) admission.

In all patients (100%), the clinical improvement at discharge was associated with the disappearance of the previous LUS findings and with a lower US score.

The US score at discharge was weakly associated with the clinical score at discharge (rho = 0.30; 95%CI = 0.07–0.51; *p* = 0.001).

The mean length of hospitalization was 4.6 ± 2.0 days. The length of hospital stay (coef. = 0.3; 95% CI = 0.1–0.5; standard error = 0.09; t = 3.7; *p* < 0.0001; R2 = 0.13) correlates with LUS score.

## 4. Discussion

The current guidelines of the American Academy of Paediatrics indicate that the diagnosis of bronchiolitis is completely based on medical history and a physical examination in children from 1 month through 23 months of age [4]. Even if LUS does not belong to the recommendations, many studies have demonstrated that a specific sonographic pattern could be related to the clinical course of the disease. In particular, the main findings are B-lines and their distribution, consolidations in the peripheral sub-pleural area and bilateral involvement of the intercostal spaces.

This study validates a previously published ultrasound score for bronchiolitis by Basile et al. [8] and highlights the correlation between LUS findings and the need for supplemental oxygen and hospital stay duration.

Caiulo et al. performed the first study that investigated US findings in bronchiolitis, showing that some lung abnormalities can be appreciated only with sonography and are not revealed by CXR. They speculated that LUS could become the routine imaging modality for this group of patients [13]. Similarly, Jaszczolt et al. point out the utility of LUS in comparison with CXR, considering the main advantages of sonographic imaging: absence of radiations, short time of examination, higher sensitivity in the evaluation of pleural effusion, small consolidations and signs of interstitial involvement in patients with bronchiolitis [14]. In recent decades, many studies suggested that LUS findings in children with bronchiolitis correlate with disease severity [15,16].

An observational prospective study by Zoido Garrote et al. demonstrated a moderate correlation between LUS findings at hospital admission and the severity of the clinical course, confirming also the higher involvement of the posterior paravertebral and subscapular areas as main severity indexes [17].

Conversely Taveira M et al. failed to demonstrate a correlation between LUS findings and the clinical course of bronchiolitis, even if they found a positive correlation between the number of involved intercostal spaces by the white lung appearance and the length of oxygen therapy [18].

Recently, a prospective multicenter observational cohort study has demonstrated that LUS could predict the need of respiratory support in a group of 145 infants <6 months with bronchiolitis. Moreover, they confirmed our consideration, showing a poorer prognosis in infants having consolidations >1 cm with an independent relative risk (RR) for respiratory support of 2.5 [CI 95%:1.6–4] [19].

Finally, a study performed in children with clinical bronchiolitis demonstrated the good accuracy of LUS in discriminating children with uncomplicated bronchiolitis from those with concomitant bacterial pneumonia [20,21].

All these works share important results with our study and demonstrate how the LUS score can be considered a useful tool in managing bronchiolitis. Our study confirms previous results about the usefulness of LUS to predict the need for oxygen supplementation in infants with mild symptoms. Finally, we speculate that the LUS score can be used to decide the most appropriate clinical setting, mostly in the early phase of bronchiolitis.

We are aware of some limitations of this study. Firstly, ultrasonography is an operator-dependent technique and in our trial was performed by an experienced physician (M.M.), so the same result might not be extended to all paediatricians. Secondly, none of the included patients required intensive care and our results might not be applied to critical settings. Thirdly, especially in the first six months of life, a complete normal A-line pattern is rarely seen and a diffuse pattern of vertical artefacts (B-lines) has been described also in healthy infants due to lung immaturity. These findings may influence the result of the LUS score that might overestimate the incidence of bronchiolitis at this critical age [22,23]. On the contrary, signs of more severe disease (pleural effusion, pleural line abnormalities or subpleural consolidations) have not been found in healthy children and they are usually the expression of an underlying infective pathology or also a congenital pulmonary malformation [24].

Further confirmations of LUS usefulness are needed to establish its role in the diagnostic and therapeutic work-up of bronchiolitis. A more comprehensive evaluation of patients clinically diagnosed with bronchiolitis might also benefit from an ultrasound evaluation of the diaphragm: its dysfunction could demonstrate an increased respiratory load earlier and prevent the progression toward respiratory failure [25].

Future studies, also performed into an ICU setting and into a wider population, may demonstrate the relationship between sonographic findings and the need of hospitalization or oxygen therapy.

## 5. Conclusions

Lung ultrasound correlates with the clinical progression of acute bronchiolitis in infants.

LUS could ameliorate the diagnostic and therapeutic management of bronchiolitis, considering as main advantages its safety, absence of invasiveness, short time of examination, low cost and real-time findings.

We speculate that a wider use of LUS and further evidence of its role in the evaluation and outcome prediction of infants with bronchiolitis could reduce the socio-economic burden of bronchiolitis, preventing unneeded hospitalization as a major component of the in-patient health care costs.

## Figures and Tables

**Table 1:** Clinical score.

Clinical Score	0	1	2	3
Respiratory rate	<50 rpm	50–60 rpm	61–69 rpm	>70 rpm
Signs of dyspnea	Normal feeding	Difficulties in feeding	At least 2 signs: difficulties in feeding, perioral cyanosis, agitation	At least 2 signs: cyanosis, interruption of feeding, drowsiness
Use of accessory respiratory muscles	None	Subcostal or intercostal retractions	At least 2 signs: subcostal retractions, intercostal retractions, substernal retractions, nasal flaring	At least 3 signs: subcostal retractions, intercostal retractions, substernal retractions, suprasternal retractions, supraclavicular retractions, nasal flaring
Signs of abnormal auscultation	Normal breathing	Only 1 sign between end-expiratory wheeze or crackles	Expiratory wheeze and/or crackles	At least 1 sign: Inspiratory and expiratory wheeze, diminished breath sounds

Results: healthy child—score 0; mild bronchiolitis—score 1–4; moderate bronchiolitis—score 5–8; and severe bronchiolitis—score 9–12.

**Table 2:** Ultrasound score.

US Score.		0	1	2
Anterolateral data		Normal lung sliding with A-linesAbsence or limited presence of B-lines	Diffuse, inhomogeneous interstitial syndrome with confluent, multiple B lines and spared areas	Diffuse, inhomogeneous interstitial syndrome and/or subpleural lung consolidations
Paravertebral/posterior data	Interstitial syndrome	Absent or individual B-lines	Focal, multiple B-lines	Confluent, multiple B-lines
	Extension on interstitial syndrome	Bilateral involvement of 0–6 intercostal spaces	Bilateral involvement of 6–12 intercostal spaces	Bilateral involvement of >12 intercostal spaces
	Presence of subpleural consolidations	Absent	Subcentimeter-subpleural lung consolidation (diameter < 1 cm)	Subpleural lung consolidation with diameter > 1 cm

Results: normal LUS pattern—score 0; mild bronchiolitis—score 1–3; moderate bronchiolitis—score 4–6; and severe bronchiolitis—score 7–8.

**Table 3:** Lung Ultrasound score and clinical progression outcomes.

	Coefficient	95%CI	*p*-Value
Clinical score at admission	rho = 0.23	0.02–0.43	0.036
Clinical score at discharge	rho = 0.30	0.07–0.51	0.001
Need for supplemental oxygen	OR = 2.2	1.5–3.3	<0.0001
Duration of oxygen supplementation	coef. = 0.01	−0.01–0.03	0.363
Length of hospital stay	coef. = 0.3	0.1–0.5	<0.0001
